# Active control of electromagnetic radiation through an enhanced thermo-optic effect

**DOI:** 10.1038/srep08835

**Published:** 2015-03-09

**Authors:** Chong Sheng, Hui Liu, Shining Zhu, Dentcho A. Genov

**Affiliations:** 1 National Laboratory of Solid State Microstructures & School of Physics, Collaborative Innovation Center of Advanced Microstructures, National Center of Microstructures and Quantum Manipulation, Nanjing University, Nanjing 210093, China; 2 Center for Applied Physics Studies, Louisiana Tech University, Ruston, Louisiana 71270, USA

## Abstract

The control of electromagnetic radiation in transformation optical metamaterials brings the development of vast variety of optical devices. Of a particular importance is the possibility to control the propagation of light with light. In this work, we use a structured planar cavity to enhance the thermo-optic effect in a transformation optical waveguide. In the process, a control laser produces apparent inhomogeneous refractive index change inside the waveguides. The trajectory of a second probe laser beam is then continuously tuned in the experiment. The experimental results agree well with the developed theory. The reported method can provide a new approach toward development of transformation optical devices where active all-optical control of the impinging light can be achieved.

Controlling the propagation of light is an important research topic in optics. The light propagation in a dielectric medium is primarily determined by the refractive index or dielectric constant of the material. At optical frequencies, however, the naturally existing materials have a rather narrow range of refractive indexes which limits their applicability to manipulate light. Recently, man-made composite materials commonly referred to as metamaterials have been proposed to dramatically extend the range of magnetic and dielectric properties of the materials existing in nature. The metamaterials are composed of large number of subwavelength in size structured elements and have been used as negative refractive index materials^1^,^2^, zero-index materials^3^,^4^, materials with high anisotropy^5^, etc. By tuning the sizes and shapes of the structural elements, it is also possible to change the spatial profile of the effective refractive index and thus control the light trajectory inside the metamaterial. A large variety of transformation optics^6^,^7^ (TO) devices based on such inhomogeneous metamaterials have been proposed including invisibility cloaks^8^,^9^,^10^,^11^, illusion optics^12^,^13^,^14^, Luneberg lens^15^, photonic black holes^16^,^17^,^18^,^19^, nanofocusing plasmonics^20^,^21^,^22^, etc. To facilitate such optical phenomenon, systems based on split-ring resonators^5^,^23^,^24^, porous silicon wafer^25^,^26^,^27^, multilayers^28^,^29^,^30^,^31^,^32^,^33^, graded lithography^15^, inhomogeneous waveguide^34^,^35^, electric-controlled graphene^36^, mixture solutions^37^,^38^, macroscopic crystal^39^,^40^,^41^,^42^ have been studied. All these approaches, however, are static which is the optical effects are pre-set and cannot be tuned or controlled once the system is physically created. On the other hand, use of nonlinear medium can provide dynamic changes in the refractive index profiles through variations in the intensity of a control laser beam^43^,^44^,^45^,^46^,^47^.

In this work, we propose an enhanced thermo-optic effect facilitated by a Fabry-Perot (FP) cavity to achieve measurable light-guiding outcomes. In the thermo-optic process, light is absorbed inside the medium, increasing its temperature which changes it refractive index. To enhance this effect a silver/polymer/silver waveguide is designed and fabricated. When a pump laser beam is incident on the system, FP resonances are excited increasing the light absorption and thus enhancing the thermo-optic effect. Furthermore, the Gaussian energy density profile of the pump laser spot produces an inhomogeneous index change in the polymer. Concurrently, a probe beam incident on the inhomogeneous refractive index experiences deflection with a deflection angle proportional to the energy density of the pump laser. The experimental results match the developed analytical theory and show that this optical system can act as a lens with a tunable focal length. The reported method offers a new approach toward light-controllable transformation optical devices.

## Results

### Experiment description

**Figure 1:**
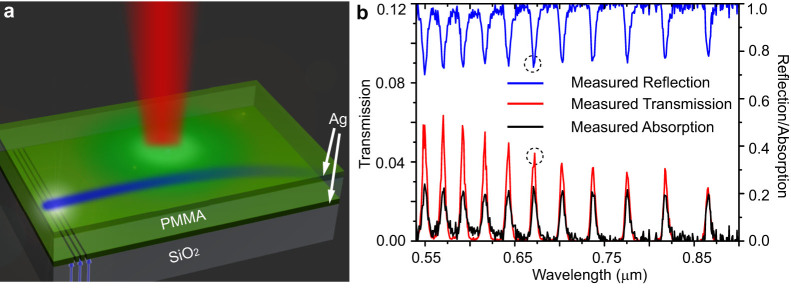
Schematics and optical characterization of experimental sample. (a) A PMMA layer is sandwiched between two sliver layers to form a Fabry-Perot cavity. The probe laser beam (blue) propagates inside the waveguide and is excited by grating drilled in the bottom silver layer. A separate control laser is incident normally to the sample and induced spatially inhomogeneous nonlinear refraction index change. (b) The measured transmission (T), reflection(R) and absorption (A) spectra of a normally incident light on the waveguide. The spectra clearly shows the FP resonances in the system with the dashed circle corresponding to the wavelength of the control laser beam (671 nm).

A schematic of the experiment sample and relevant phenomenon is illustrated in figure 1(a). In the experiment a planar silver/PMMA/silver waveguide is used to guide a probe laser beam. A second Gaussian shape control/pump beam is incident normally on the waveguide and induces a change in the refractive index of the PMMA layer due to thermal heating. The maximal effect is achieved if the pump laser wavelength is tuned to a particular Fabry-Perot (FP) resonance of the waveguide, note that the silver/PMMA/silver waveguide acts as a cavity for external radiation. The measured absorption, transmission and reflection spectrum of the cavity showing the FP resonances are given in figure 1(b). For the given geometry and frequency range of operation the absorbance can be as high as 20%. In the optical spectral range the PMMA is a weak absorber and without a cavity will absorb less than 0.4% of the impinging light for the given thickness (4.8 microns) of the polymer. The enhanced absorption in structured waveguide considered here can be used to dramatically enhance the thermo-optic effect in the polymer.

To study the thermo optical effect in the experiment, a probe laser beam with wavelength of 457 nm is coupled into the silver/PMMA/silver waveguide through a grating with period 310 nm drilled on the silver film between the substrate and the PMMA layer (see figure 1(a)). A second control Gaussian beam with width *σ* = 24 *μm* illuminates the cavity from above and is tuned to the 671 nm FP resonance, the dashed circles in figure 1b, thus facilitating the nonlinear change of the refractive index of the PMMA. In order to directly observe the trajectory of the probe beam inside the waveguide, the PMMA layer is doped with Eu^3+^. As the probe beam propagates inside the PMMA layer, it excites the Eu^3+^ atoms that then emit fluorescence radiation at 610 nm wavelength. This radiation is recorded and used to study the effect of the thermo-optic nonlinearity on the propagation of the probe beam.

**Figure 2:**
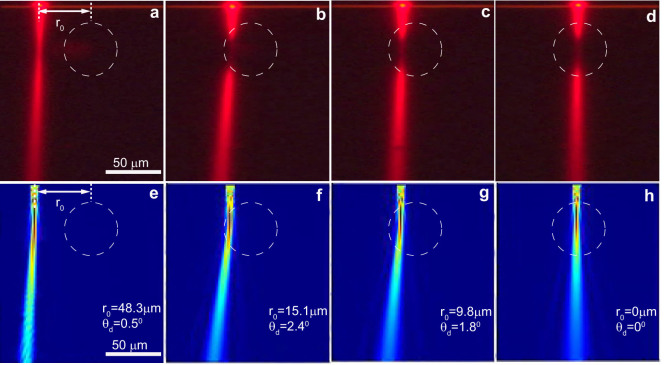
The deflection angle vs. impact parameter. (a) – (d) The measured light trajectories (fluorescence imaging) for decreasing impact parameter *r*_0_. In the experiment the power of the control laser is fixed at 2.8 W. In the figures the spatial extend of the input Gaussian beam is identified with dashed circles with radius equal to the beam width *r* = *σ*. (e) – (h) The numerically calculated beam propagation in the system closely resemble the experimental measurements, thus indirectly validating the developed model for the thermo-optical effect given by equation (1).

The deflection angle of a paraxial ray incident on an ordinary optical lens is given as *θ_d_* = tan^−1^(*r*_0_/*f*) ≈ *r*_0_/*f*, where *f* is the focal length and *r*_0_ is the impact parameter or the distance of the incident ray to the optical axis. This simple relationship can be obtained by considering only the refraction of the incident beam at the lens interfaces. For inhomogeneous optical media, however, the dependence of the deflection angle on the impact parameter can be rather complex. Still knowledge of the *θ_d_*(*r*_0_) dependence can serve as a powerful tool to quantify the effect of the inhomogeneity on the ray trajectories. In the experiment, the probe beam is excited by the grating, propagates toward the center of the nonlinearity produced by the pump beam and concurrently is deflected by the angle *θ_d_*. The recorder ray trajectories for four different impact parameters are shown in figure 2. In figure 2(a), the incident beam has an impact parameter *r*_0_ = 48.3 *μm* that is large compared to the extent of the enhanced nonlinearity region (given by the pump beam width *σ* = 24 *μm*). Correspondingly the bending effect is weak. As the impinging light beam approaches the center of inhomogeneity (the “lens”), the deflection angle increase and reaches a maximum value *θ_d_* = 2.4° for *r*_0_ = 15.1 *μm* (see figure 2(b)). However, further decrease in the impact parameter results in a near linear decrease of the deflection angle as seen in figure 2(c) and (d). This behavior is similar to that of an ordinary concave optical lens.

To provide a better physical description of the experimental results, we consider the Largangian formalism for the ray trajectories in the system coupled with a phenomenological model of the thermo-optic effect.

### Thermo-optic effect

The refractive index of the PMMA under inhomogeneous thermal conditions is given as 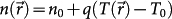, where *q* = *dn*/*dT* is the thermo-optic coefficient, 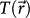 is the local temperature, *T*_0_ is the ambient temperature (away from the heat source) and *n*_0_ is the refractive index at the ambient temperature. The PMMA has a large negative thermo-optic coefficient^48^ *q* ≈ −1.15 × 10^−4^ K^−1^, and ambient refractive index *n*_0_ = 1.499 (at the probe laser beam wavelength of 457 nm). The local temperature is obtained from the inhomogeneous heat equation 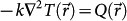, where *Q* is the heat delivered by the control laser and *k* is the thermal conductivity of the PMMA. Under the specific conditions of the experiment, a waveguide thickness *d* that is substantially smaller compared to the width of the control Gaussian beam *σ*, we have (see Methods):
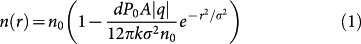
where, *P*_0_ is the total laser power and *A* is the absorption coefficient.

**Figure 3:**
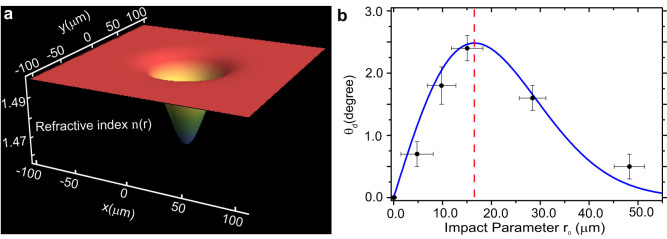
Inhomogeneous refractive index and deflection angle. (a) The induced refractive index profile calculated using equation (1) and fixed controlling laser power at 2.8 W. (b) The deflection angle vs. the impact parameter calculated based on equation (2) (blue line) is found to be in excellent correspondence with the experimental data (symbols). The red dashed line corresponds to the optimal impact parameter 
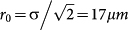
.

The refractive index profile according to equation (1) is shown in figure 3 (a). The heating due to a control laser with *P*_0_ = 2.8 W reduces the refractive index of the PMMA at the center of illumination by 2.86%. This is a significant nonlinear change which corresponds to a nonlinear refractive index *n*_2_ = *d*|*q*|*A*/12*k* = 2.76 × 10^−7^ cm^2^*W*^−1^ and exemplifies the great potential of the thermo-optic effect as a mean of controlling light in our system. Based on the refractive index profile equation (1), we have simulated the light propagation of the probe beam in the waveguide using commercial FDTD software (Lumerical Solutions, Inc.). The numerical results are depicted in figure 2 (e–h), showing a good agreement with the experimental results (see figure 2 (a–d)), thus validating the developed thermo-optic model given by equation (1).

### Ray trajectories and angle of deflection

The ray trajectories in the central symmetric refractive index equation (1) trigged by the PMMA's thermo-optic effect, are described by the Lagrangian^16^ 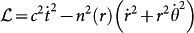, where the derivatives are taken over an arbitrary affine parameter. By solving the Euler-Lagrange equations it is easy to obtain a first integral of motion and accordingly the deflection angle as function of the impact parameter distance *r*_0_ (see Methods):
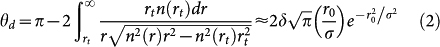
where the turning point *r_t_* is related to the impact parameter according to *r*_0_ = *r_t_n*(*r_t_*)/*n*_0_, and 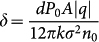. The theoretical result equation (2) is in excellent agreement with the experiment as shown in figure 3(b). For small impact parameters 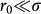, we have a linear increase of *θ_d_* with the increase in the impact parameter similarly to what one expects for an ordinary concave lens with an effective focal length of 
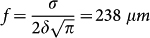
. With the further increase in the impact parameter the deflection angle reaches a maximum for 
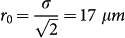
, and then exponentially decreases.

**Figure 4:**
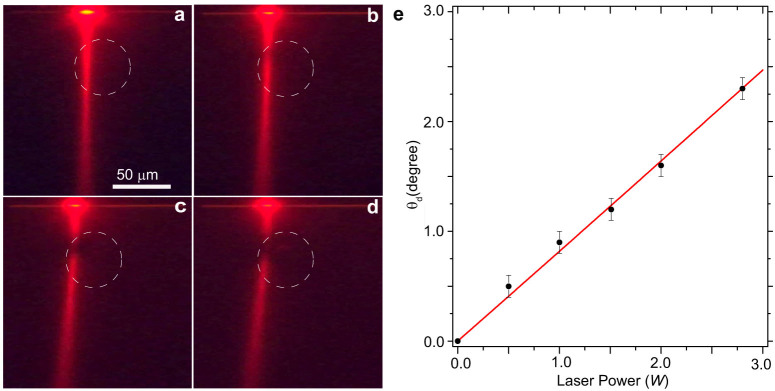
The deflection angle vs. laser power. (a–d) The experimentally measured light trajectories (fluorescence imaging) vs. the total power of the control laser at a fixed impact parameter (*r*_0_ = 13.9 μm). The laser power is (a) 0 W (*θ_d_* = 0), (b) 1 W (*θ_d_* = 0.9°), (c) 2 W (*θ_d_* = 1.6°), and (d) 2.8 W (*θ_d_* = 2.3°). (e) The experimentally measured deflection angle (symbols) vs. the input power is in excellent correspondence with the theory equation (2) (red line).

The theory predicts a liner dependence of the deflection angle with the control laser beam power *θ_d_* ∝ *δ* ∝ *P*_0_. To study this effect we fix the impact parameter at *r*_0_ = 13.9 *μm*, and vary the input power of the control beam. A comparison between the experiment and theory is shown in figure 4. As expected the deflection angle increases linearly with the input power and closely follows the theoretical line with a slope of 0.84 deg/W. For the available experimental range, saturation in the nonlinear response is not being observed which exemplified the potential to achieve even stronger nonlinear effects in the proposed waveguide configuration.

The inhomogeneous guiding effect can be controlled by changing the intensity and/or shape of the absorbed radiation. Employing control illumination through pre-set apertures or by using vortex beams with spatial light modulator (SLM)^49^,^50^, in principle, we can produce a large variety of inhomogeneous index profiles. A direct control of heat flow, as recently demonstrated in metamaterials^51^,^52^, can also be employed to achieve similar functionalities. We must also note that our technique can be modified to operate with other nonlinear materials. Polymers such as poly(methylmethacrylate), polystyrene, and polycarbonates are of special interest since in general they have higher thermo-optical coefficients compared to non-organic materials^53^,^54^,^55^.

## Discussion

We have experimentally demonstrated and theoretically modeled the guiding and deflection of light due to an enhanced thermo-optic effect in transformation optical waveguides. In the experiment the thermo-optic effects is due to the resonant absorption in the PMMA of a control laser beam. The inhomogeneous intensity (Gaussian beam) of the control laser induces a radially varying refractive index profile in the waveguide which allows for active manipulation (deflection) of a secondary probe beam. For small impact parameters the systems behaves as a concave lens with a tunable effective focal length. In our system a direct control of the heat flow or/and illumination through pre-set apertures can be utilized to develop multi-physics transformation optical devices that guide light in a pre-determent way.

In this work, we just simply demonstrate the basic idea how to employed thermal effect to produce gradient index medium and realize optical control of light propagation inside PMMA waveguide. In the future research, this method can be used to manipulate the light propagation to follow a really transformed path with meaningful applications.

## Method

### Sample fabrication

First a 40 nm silver film is sputtered on a glass substrate. A grating with 310 nm period, used to couple light into the planar waveguide, is drilled on the sliver film with focused ion beam (FEI Strata FIB 201, 30 keV, 150 pA). A PMMA resist for certain solubility (1.5 g PMMA powder dissolved in 10 mL toluene) mixed with Eu^3+^ is spin coated on the sample and dried in an oven at 70°C for 2 h. The thickness of the polymer layer is about 4.8 μm. The Eu^3+^ is added for the purpose of fluorescence imaging of the ray trajectories inside the waveguide. Finally, a 28 nm cladding sliver layer is deposited on the sample using electron beam evaporation thus forming the Fabry-Perot resonator.

### Theoretical formation of the thermo-optic effect

We consider the steady state heat equation with a cylindrically symmetric heat source (Gaussian beam):
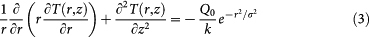
where *T* is the laser induced temperature profile inside the waveguide, *k* = 0.25 W/m. K is the thermal conductivity of the PMMA and *Q*_0_ is maximum heat density delivered by the control laser. If the waveguide thickness *d* is substantially smaller than the width of the Gaussian beam *σ*, i.e. 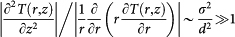, then equation (3) can be reduced to 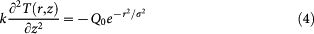
This equation has explicit solution of the form
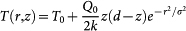
where we have enforced the boundary condition *T*(*r*, 0) = *T*(*r*, *d*) = *T*_0_, with *T*_0_ being the ambient temperature. Finally, introducing the average temperature across the waveguide 
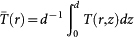
 we obtain the nonlinear refractive index of the PMMA as:
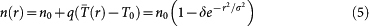
where 
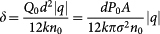
 and *P*_0_ is the total power of the control laser.

### Ray trajectories and angle of deflection

The ray trajectories in the centrally symmetric media given by the refractive index equation (5), are described by the Lagrangian^16^ 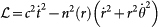, where the derivatives are taken over an arbitrary affine parameter. Assuming a planar motion, the Euler-Lagrange equations coupled with the null-geodesic condition 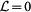, lead to the first integral of motion
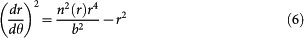
where *θ* is azimuthal angle, and *b* = *n*_0_*r*_0_ is related to the impact parameter *r*_0_. Introducing the non-dimensional radial coordinate *ζ* = *r*/*σ*, the ray trajectory follows from equation (6) as
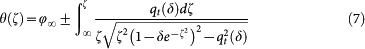
where 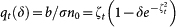 and *ζ_t_* = *r_t_*/*σ* is the turning point. The above integral does not have an analytical solution. However, the thermo-optic effect is weak and by expanding the integrant with respect to 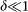 and keeping only the first two terms in the expansion we obtain
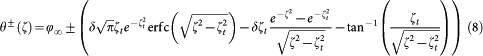
where erfc is the complimentary error function. The deflection angle as function of the impact parameter distance *r*_0_ thus follows as
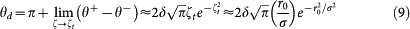
where we have used that *ζ_t_* ≈ *b*/*σn*_0_ = *r*_0_/*σ*, for 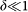.
